# Novel recombinant bovine papular stomatitis virus expressing foot-and-mouth disease virus-like particles elicits neutralizing antibodies in cattle

**DOI:** 10.1099/jgv.0.002344

**Published:** 2026-11-02

**Authors:** Herman M. Chambaro, Gustavo Delhon, María Cruz Miraglia, Sabrina Galdo-Novo, Ana Taffarel, Byung-Joon Seung, Sushil Khatiwada, Daniel Mariano Pérez-Filgueira, Daniel L. Rock

**Affiliations:** 1 Department of Pathobiology, College of Veterinary Medicine, University of Illinois at Urbana-Champaign, Urbana, IL, USA; 2 School of Veterinary Medicine and Biomedical Sciences and Nebraska Center for Virology, University of Nebraska-Lincoln, Lincoln, NE, USA; 3 Instituto de Virología e Innovaciones Tecnológicas, CICVyA, INTA-CONICET, Buenos Aires, Argentina; 4 WOAH FMD Reference Laboratory, SENASA, Buenos Aires, Argentina

**Keywords:** cattle immunization, FMD virus (FMDV), foot-and-mouth disease, recombinant bovine papular stomatitis virus, vaccine, virus-like particles

## Abstract

Foot-and-mouth disease (FMD) remains a major burden in endemic regions, where inactivated vaccines are constrained by cost, short duration of immunity, cold-chain dependence and high-containment manufacturing. Here, we engineered a recombinant bovine papular stomatitis virus (rBPSV) expressing the FMD virus (FMDV) A24 Cruzeiro capsid precursor P1-2A together with an attenuated 3C protease (3C^proL127P^). Infection of ovine foetal turbinate cells resulted in FMDV capsid protein expression and assembly of 25–30 nm icosahedral virus-like particles (VLPs). Intramuscular immunization of two BPSV-seropositive calves with rBPSV (10^7^ TCID_50_ on days 0, 21 and 35) induced anti-FMDV humoral responses. By day 28, liquid-phase blocking ELISA titres exceeded laboratory-defined protective threshold (≥1.9 log_10_), and homologous neutralizing titres against A24 Cruzeiro surpassed the threshold associated with protection (≥1.36 log_10_). Intra-serotypic cross-neutralization was observed against A/Argentina/2001, whereas no neutralization was detected against heterologous serotype O_1_ Campos. Pre-existing anti-BPSV antibodies did not prevent induction of neutralizing responses. These findings establish the first proof of concept that BPSV can serve as a cattle-adapted vector platform for delivery of FMDV VLPs.

## Data Availability

The authors confirm that the data supporting the findings of this study are available within the article.

## Introduction

Foot-and-mouth disease (FMD) is a transboundary animal disease that continues to impose major constraints on global livestock production and international trade [1, 2]. The causative agent, FMD virus (FMDV; genus *Aphthovirus*, family *Picornaviridae*), is a small, non-enveloped, positive-sense RNA virus with an ~8.5 kb genome that is translated into a single polyprotein, which is proteolytically processed into structural and non-structural proteins [3]. The viral capsid comprises 60 copies each of VP1, VP2, VP3 and VP4, produced from the P1-2A precursor by the viral 3C protease (3C^pro^) and assembled via protomer and pentamer intermediates into icosahedral particles displaying conformational neutralizing epitopes [3]. Coordinated expression of P1-2A with controlled 3C^pro^-mediated processing enables efficient formation of genome-free virus-like particles (VLPs) that preserve native protective antigenic structure while avoiding the requirement to propagate infectious FMDV [4].

Vaccination is central to FMD control and can support regional elimination when applied at scale [5, 6]. However, conventional chemically inactivated FMDV vaccines remain poorly suited to endemic settings: immunity is often short-lived, especially in young cattle; cross-protection among serotypes is limited; antigen integrity is compromised by thermal instability and acid sensitivity, requiring strict cold-chain logistics; and production depends on large-scale propagation of live FMDV in costly, high-containment (BSL-3) facilities [7]. These limitations contribute to suboptimal coverage and recurrent outbreaks, highlighting the need for safe, scalable vaccine platforms better suited to resource-limited settings.

VLPs are attractive next-generation FMD vaccine candidates because they preserve the native conformational neutralizing epitopes of the viral capsid while remaining non-infectious [8]. Recombinant FMDV VLPs have been produced using bacterial, insect, mammalian cell and nucleic acid-based expression systems, with several platforms demonstrating immunogenicity and, in some cases, protection following experimental challenge in cattle [7, 9]. However, most rely on ex vivo antigen production, purification and formulation before vaccination, and their broader application has been constrained by platform-specific limitations, including restricted cargo capacity, variable antigen expression, inconsistent particle yields and the need for repeated administration [9]. A replication-competent, cattle-adapted vector capable of producing FMDV VLPs directly within the target host therefore represents a distinct VLP delivery strategy that integrates antigen production and delivery into a single vaccine platform.

Bovine papular stomatitis virus (BPSV), a parapoxvirus ubiquitous in cattle and associated with absent or mild, self-limiting disease, is a promising viral vector platform [10]. Its favourable attributes include strict host-species restriction, weak and transient anti-vector immunity, persistence in the host, efficient horizontal transmission with high reinfection potential and substantial foreign-gene cargo capacity [10–15]. Collectively, these characteristics support repeated administration, manufacture under BSL-2 conditions and the potential development of Differentiating Infected from Vaccinated Animals-compatible vaccination strategies for FMD control in resource-limited endemic settings. Moreover, the natural ability of BPSV to reinfect cattle raises the possibility that repeated exposure to a recombinant vector could reinforce antigen-specific immune responses, although this hypothesis remains to be experimentally evaluated.

Here, we evaluated a recombinant BPSV (rBPSV) expressing FMDV VLPs. We show that BPSV supports *in vitro* production of FMDV VLPs and induces neutralizing antibody responses in BPSV-seropositive calves, providing proof of concept for the further development of BPSV as a cattle-adapted VLP vaccine platform for FMD and potentially other veterinary pathogens.

## Methods

### Cells and viruses

Ovine foetal turbinate (OFTu) cells were maintained in minimum essential medium supplemented with 10% FBS and 1× penicillin–streptomycin (100 U ml^−1^ penicillin and 100 µg ml^−1^ streptomycin) at 37 °C in a humidified atmosphere containing 5% CO_2_. WT BPSV (C5 strain) [10] and the rBPSV described below were propagated and titrated on primary OFTu cells. Virus titres were determined by the Reed–Muench method and expressed as 50% tissue culture infectious dose (TCID_50_)/ml.

### Construction of rBPSV expressing FMDV VLPs

To generate the rBPSV transfer vector, the FMDV A24 Cruzeiro (accession no. AY593768) capsid precursor P1-2A (VP4, VP2, VP3, VP1 and 2A) and the viral 3C^pro^ coding sequences were synthesized (GenScript, Piscataway, NJ, USA). Because the WT 3C^pro^ can cleave host cellular proteins, including Histone H3, SAM68, NEMO, eIF4AI and eIF4GI, during capsid precursor processing, resulting in cellular toxicity and reduced recombinant protein production, the previously described L127P (3C^proL127P^) substitution was introduced into the protease. This mutation has been shown to reduce protease-associated cytotoxicity while maintaining efficient processing of the P1-2A capsid precursor [4].

The P1-2A and 3C^proL127P^ coding sequences were assembled as a single ORF (P1-2A-3C^proL127P^) under the control of the vaccinia virus vv7.5 early/late promoter. The resulting expression cassette was cloned into a pUC57 transfer plasmid containing 768 bp (left flank) and 871 bp (right flank) sequences homologous to the regions flanking the BPSV024 gene, which encodes an NF-κB inhibitory protein [10], to facilitate site-specific insertion by double-crossover homologous recombination. An EGFP reporter gene under the control of the vaccinia virus vv13.5 promoter was incorporated into the recombination cassette to facilitate identification and purification of recombinant viruses. The integrity of the transfer plasmid was confirmed by AsiSI/AscI restriction enzyme analysis and Sanger sequencing.

OFTu cells were infected with parental BPSV C5 at a m.o.i. of 1 and incubated for 1 h before transfection with 2.5 µg of the transfer plasmid using Lipofectamine 3000 Transfection Reagent (Thermo Fisher Scientific, Waltham, MA, USA) according to the manufacturer’s instructions. At 72 h post-infection (hpi)/transfection, cells and culture supernatants were harvested and subjected to three freeze–thaw cycles to release viral progeny. rBPSV was initially enriched through four sequential rounds of limiting dilution, during which EGFP fluorescence was used to identify recombinant virus. The enriched virus population was subsequently purified by four rounds of plaque purification to obtain a clonal recombinant virus. Correct insertion of the P1-2A-3C^proL127P^ expression cassette into the BPSV024 locus was confirmed by PCR using primers spanning the 5′ and 3′ recombination junctions (forward: 5′-AAATGTAATGCGCCTTTCCCAAC-3′; reverse: 5′-GATCTCGGCGATCCTGTTTTTTATG-3′), followed by Sanger sequencing of the PCR amplicons.

### VLP production, purification and characterization

OFTu cells were infected with rBPSV-FMDV^P1-2A-3CproL127P^ at an m.o.i. of 0.1 to produce FMDV VLPs. At 24 and 72 hpi, cells were harvested and subjected to three freeze–thaw cycles to release intracellular VLPs. Cell lysates were clarified by low-speed centrifugation (4,000 ***g***, 20 min, 4 °C), and the resulting supernatants were sequentially filtered through 0.45 µm and 0.22 µm membrane filters. VLPs were concentrated by precipitation with 7% (wt/v) polyethylene glycol (PEG 6000) and subsequently purified by ultracentrifugation through a 20% (wt/v) sucrose cushion (100,000 ***g***, 4 h, 4 °C). The VLP-containing pellets were resuspended in Tris-NaCl-EDTA buffer and analysed by SDS-PAGE followed by Coomassie Brilliant Blue staining. The morphology and size of purified VLPs were assessed by transmission electron microscopy (TEM) following negative staining with 1% (wt/v) uranyl acetate.

### Immunogenicity of rBPSV-FMDV^P1-2A-3CproL127P^ in cattle

Two calves (4–5-month-old) shown to be BPSV-seropositive by an in-house indirect ELISA were vaccinated intramuscularly with 10⁷ TCID_50_ of rBPSV-FMDV^P1-2A-3CproL127P^ on days 0, 21 and 35. Sera collected on days 0, 7, 14, 21, 28, 35, 42, 49 and 56 were analysed for FMDV-specific humoral responses using complementary assays targeting distinct aspects of the antibody response. Antibodies recognizing the immunodominant VP1 G-H loop (residues 135–160), which contains a major neutralizing antigenic site of FMDV, were quantified by indirect ELISA using an equimolar mixture of three overlapping synthetic peptides spanning the G-H loop (Table 1). Each peptide was synthesized with a C-terminal cysteine to permit covalent immobilization on maleimide-activated microplates (Thermo Fisher Scientific, Rockford, IL, USA).

**Table 1.** Overlapping synthetic peptides spanning the FMDV A24 Cruzeiro VP1 G-H loop used as an equimolar mixture in the indirect ELISA

Peptide	Sequence	Residues
Peptide-1	TVYNGTSKY**C**	128–136
Peptide-2	NGTSKYAVGGSGRRGDMGSLAARVVKQ**C**	131–157
Peptide-7	GRRGDMGSLAARVVKQ**C**	142–157

All peptides were synthesized with a C-terminal cysteine to enable covalent coupling to maleimide-activated ELISA plates.

Antibody reactivity against native, intact FMDV capsids was measured by liquid-phase blocking ELISA (LPBE), as recommended in the WOAH Terrestrial Manual for serological evaluation of FMD vaccines [2]. Because the assay uses intact virions as the target antigen, it detects antibodies directed against multiple conformational capsid epitopes and provides an assessment of the broader anti-capsid antibody response. Functional neutralizing activity was determined by a conventional virus neutralization test (VNT) using cell culture-adapted FMDV strains in BHK-21 cells, as previously described [16]. Neutralizing antibody titres were expressed as the log_10_ reciprocal serum dilution that neutralized 50% of the virus inoculum, calculated using the Reed–Muench method [17].

To assess antigenic breadth, serum neutralization was evaluated against other viruses spanning increasing antigenic distance, including the homologous strain A24 Cruzeiro, an intra-serotypic heterologous strain (A/Argentina/2001) and a cross-serotypic heterologous strain (O_1_ Campos), consistent with established approaches for evaluating homologous and heterologous FMDV vaccine responses [18, 19]. Serological data were interpreted in accordance with WOAH guidelines [2] and laboratory-established protective thresholds defined by SENASA (WOAH Reference Laboratory, Argentina) [16, 20]. For A24 Cruzeiro and A/Argentina/2001, the expected percentage of protection (EPP) values were derived from previously established correlations between LPBE titres [20, 21] or VNT titres [22, 23] measured in vaccinated cattle at 60 days post-vaccination and challenge outcomes assessed at 90 days post-vaccination using the Protection against Podal Generalization (PPG) assay [2]. These correlations were generated from groups of 16 vaccinated cattle challenged with the homologous virus. For both LPBE and VNT, an EPP of ≥75% was used as the reference threshold for antibody titres associated with protection against homologous challenge.

## Results

### Generation of rBPSV-FMDV^P1-2A-3CproL127P^ and *in vitro* VLP production

rBPSV-FMDV^P1-2A-3CproL127P^ was successfully generated with no apparent reduction in viral replication kinetics compared with the parental BPSV C5 strain under the conditions tested (Fig. 1c). Following infection of OFTu cells with rBPSV-FMDV^P1-2A-3CproL127P^ and particle enrichment by PEG precipitation and sucrose-cushion ultracentrifugation, SDS-PAGE revealed protein bands with apparent molecular masses consistent with those expected for the FMDV capsid proteins (Fig. 1d). TEM revealed numerous non-enveloped particles with morphology consistent with FMDV VLPs, the majority measuring ~25–35 nm in diameter (Fig. 1e, f). The number of particles observed increased markedly between 24 (Fig. 1e) and 72 hpi (Fig. 1f). Collectively, SDS-PAGE and TEM findings support proteolytic processing of the FMDV capsid precursor and assembly of particles with morphology consistent with FMDV VLPs *in vitro*.

**Fig. 1.**
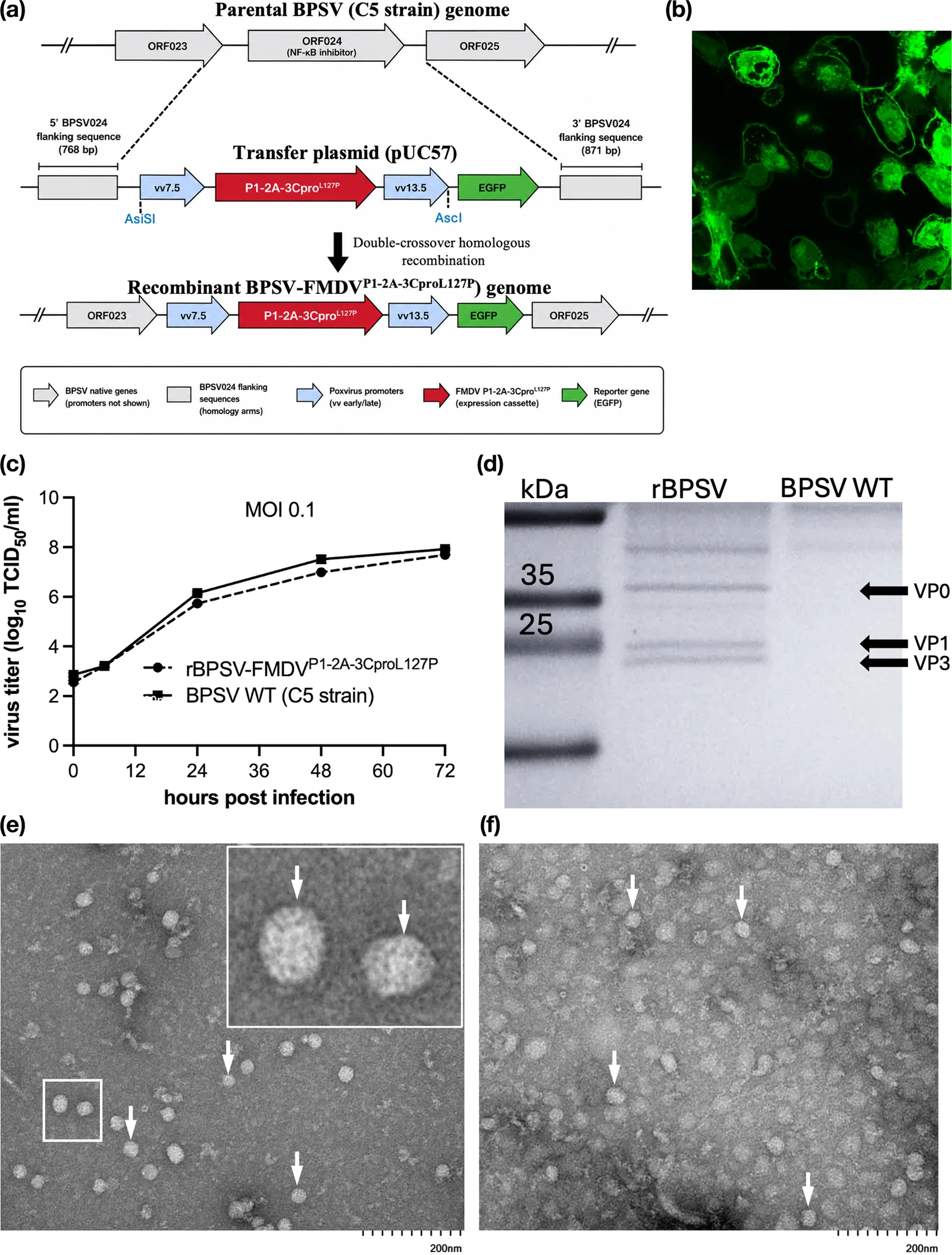
Construction and characterization of rBPSV expressing FMDV A24 Cruzeiro structural proteins and mutant (L127P) 3C^pro^ [4] (rBPSV-FMDV^P1-2A-3CproL127P^). (**a**) Schematic illustrating insertion of the FMDV expression cassette into the BPSV024 locus by double-crossover homologous recombination. The cassette comprises the vaccinia virus vv7.5 early/late promoter driving expression of the FMDV A24 Cruzeiro P1-2A-3C^proL127P^ ORF and the vv13.5 promoter driving EGFP, flanked by the left and right BPSV024 homologous recombination arms. (**b**) Fluorescence micrograph of rBPSV-FMDV^P1-2A-3CproL127P^-infected cells showing EGFP expression. (**c**) Multi-step growth kinetics of rBPSV-FMDV^P1-2A-3CproL127P^ compared to WT BPSV (strain C5) in OFTu cells infected at an m.o.i. of 0.1. (**d**) SDS-PAGE of purified VLP preparations from OFTu cells infected with rBPSV-FMDV^P1-2A-3CproL127P^ or WT BPSV, showing VP0, VP1 and VP3 (arrows). (**e, f**) TEM of negative-stained VLP preparations from infected OFTu cells harvested at 24 hpi (**e**) and 72 hpi (**f**), showing ~25–30 nm icosahedral particles consistent with FMDV empty capsids. Inset in (**e**), higher magnification. Scale bar, 200 nm.

### Humoral immunogenicity of rBPSV-FMDV^P1-2A-3CproL127P^ in BPSV-seropositive calves

Immunization of two BPSV-seropositive calves with rBPSV-FMDV^P1-2A-3CproL127P^ induced FMDV-specific humoral responses (Fig. 2a–c). No BPSV-associated lesions or other adverse clinical signs were observed in either calf throughout the study period. Antibodies to the VP1 G-H loop increased steadily after priming, peaked by day 28 and remained elevated through day 56 (Fig. 2a). Total anti-capsid reactivity measured by LPBE increased after the first boost and remained above the defined threshold associated with protection (≥1.9 log_10_) [20, 21] throughout the study period (Fig. 2b). Similarly, functional neutralizing antibody titres against homologous FMDV A24 Cruzeiro exceeded the defined protective threshold (≥1.36 log_10_) [22, 23] following the first boost (Fig. 2c).

**Fig. 2.**
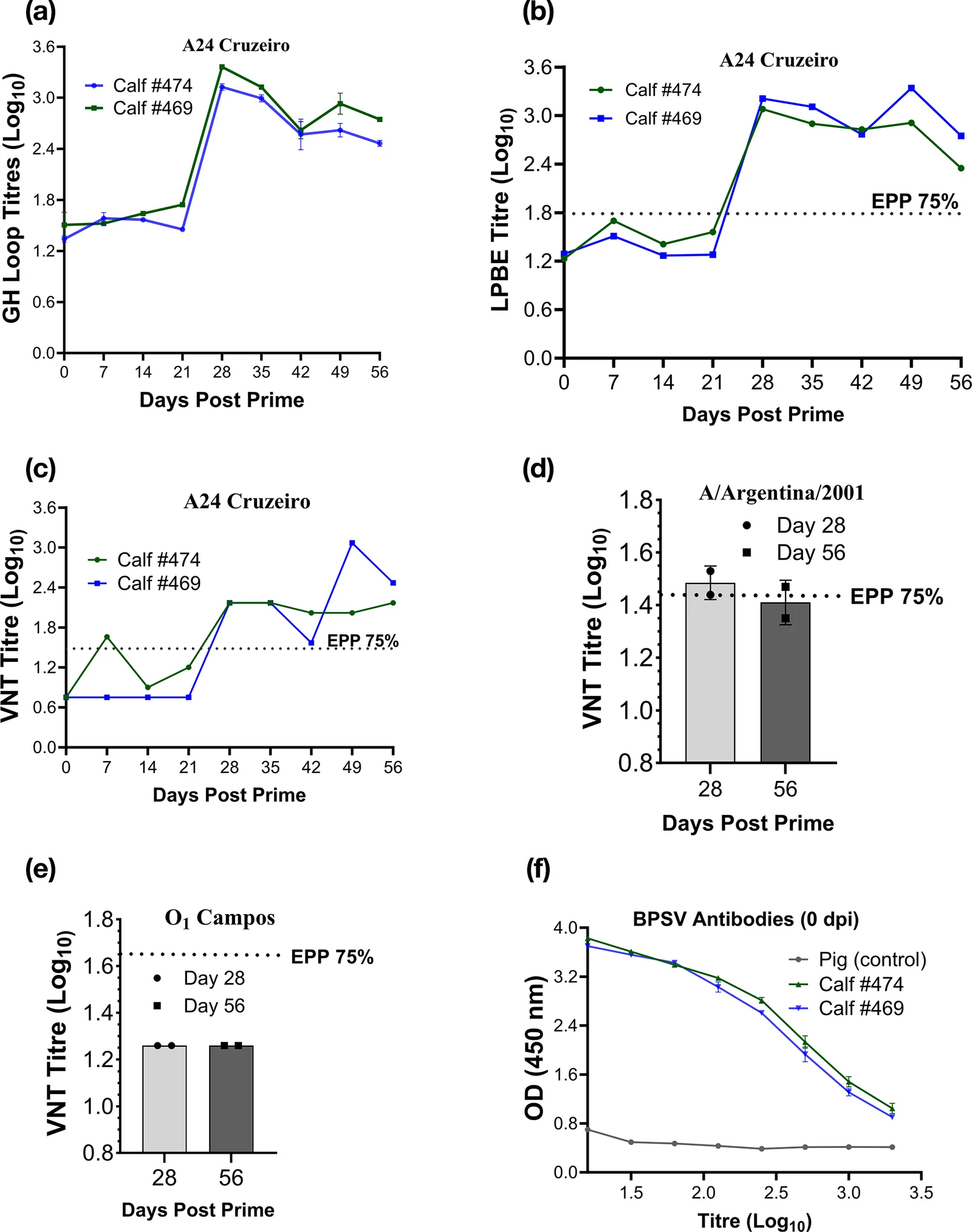
Humoral responses of rBPSV-FMDV^P1-2A-L127P3Cpro^ in BPSV-seropositive calves. Two BPSV-seropositive calves were immunized intramuscularly with 10^7^ TCID_50_ of rBPSV-FMDV^P1-2A-3CproL127P^ on days 0, 21 and 35. Sera collected on days 0–56 were analysed for FMDV-specific antibody responses. (**a**) VP1 G-H loop peptide ELISA titres (log_10_). (**b**) Total anti-capsid antibody titres measured by LPBE (log_10_); dashed line indicates the 75% EPP. (**c–e**) Neutralizing antibody titres (VNT; log_10_) against homologous A24 Cruzeiro (**c**) and heterologous strains A/Argentina/2001 (**d**) and O_1_ Campos (**e**). Dashed lines indicate the 75% EPP for A/24/Cruzeiro (VNT>1.36), A/Argentina/2001 (VNT>1.43) and O_1_ Campos (VNT>1.65). (**f**) Baseline BPSV-specific antibody titration curves for the two calves before immunization (day 0), determined by indirect ELISA. A BPSV-seronegative pig serum served as the negative control. The x-axis shows the log_10_ reciprocal serum dilution, and the y-axis shows the corresponding ELISA OD_450_ values.

Assessment of antigenic breadth demonstrated measurable cross-neutralization of the heterologous intra-serotypic strain A/Argentina/2001, with titres approaching 75% EPP threshold at day 28 before declining towards borderline levels by day 56 (Fig. 2d). In contrast, neutralization of the heterologous cross-serotypic strain O_1_ Campos was not detected at any time point (Fig. 2e). Importantly, pre-existing anti-BPSV antibodies did not prevent induction of FMDV-specific neutralizing responses following vaccination. Collectively, these data show that rBPSV-FMDV^P1-2A-3CproL127P^ induces robust, functional humoral immunity even in animals with prior exposure to BPSV and rBPSV (Fig. 2f).

## Discussion

Conventional inactivated FMD vaccines remain central to control programmes, but high cost, poor thermostability, short-lived immunity and limited heterologous protection [7, 9] underscore the need for alternative platforms. This study provides the first report that a cattle-adapted parapoxvirus can be engineered to generate high-yield FMDV VLPs capable of inducing functional immunity in cattle, even in the presence of baseline vector immunity.

The performance of rBPSV-FMDV^P1-2A-3CproL127P^ at the level of antigen production is particularly compelling. BPSV accommodated the ~4 kb P1-2A-3C^proL127P^ cassette at the BPSV024 locus without an apparent reduction in replication kinetics under the conditions tested (Fig. 1c), consistent with a previous report demonstrating successful foreign-gene insertion into the BPSV genome [10]. SDS-PAGE of purified VLP preparations revealed protein bands with apparent molecular masses consistent with the expected sizes of the FMDV capsid proteins VP0, VP1 and VP3 (Fig. 1d), while TEM demonstrated abundant non-enveloped particles (Fig. 1e, f), the majority measuring ~25–35 nm in diameter, with morphology consistent with FMDV VLPs (Fig. 1c–e). Some heterogeneity in particle size and morphology was observed, although most particles remained within the expected size range for FMDV VLPs. Together, these findings demonstrate that BPSV supports efficient processing of the FMDV capsid precursor and assembly of VLPs in infected cells, establishing proof of concept for a cattle-adapted platform capable of combining antigen production and delivery within the target host. Unlike previous FMDV VLP platforms, which rely on bacterial, insect, mammalian or nucleic acid-based expression systems requiring ex vivo antigen production [7, 9], rBPSV integrates VLP production and delivery within the target host.

Importantly, rBPSV-FMDV^P1-2A-3CproL127P^ induced functional neutralizing antibody titres against homologous A24 Cruzeiro that exceeded the defined protective thresholds (>1.36 log_10_), with LPBE titres persistently above the protective threshold (≥1.9 log_10_) (Fig. 2a–c). Based on established serological correlates [20–23], these responses are consistent with protection against homologous challenge in the PPG model. Antigenic breadth analyses showed intra-serotypic cross-neutralization against A/Argentina/2001 (Fig. 2d), with titres approaching the 75% EPP threshold at peak responses, whereas no neutralization was detected against the heterologous serotype O_1_ Campos (Fig. 2e), consistent with the serotype specificity of FMDV neutralizing antibodies [2, 18, 19]. Thus, rBPSV-FMDV^P1-2A-L127P3Cpro^ elicited both strong homologous neutralization and measurable within-serotype cross-neutralization. Vector and cassette optimization, including molecular adjuvants and modulation of viral immunomodulatory genes, may improve vaccine potency and durability of neutralizing responses.

Although anti-vector immunity can limit boosting and repeat immunization [24], BPSV is ubiquitous in cattle, and reinfection is common, consistent with weak and transient anti-vector immunity [10, 15, 25]. Accordingly, pre-existing anti-BPSV antibodies did not prevent induction of FMDV-specific neutralizing responses in vaccinated calves (Fig. 2f). Together with manufacture under BSL-2 conditions, compatibility with lyophilization and capacity for large foreign-gene insertion, the features above support BPSV as a scalable platform for repeated, multivalent vaccination in endemic settings and for broader veterinary vaccine applications. The absence of BPSV-associated lesions observed in the present study is consistent with previous reports that BPSV infection in cattle is generally mild and self-limiting [10, 12].

The present study has several limitations. First, immunogenicity was evaluated in only two calves, and no FMDV challenge was performed. Consequently, although LPBE and VNT titres reached established serological thresholds associated with protection [20–23], these responses should be interpreted as correlates of protection rather than direct evidence of protective efficacy. Second, both calves were BPSV-seropositive at enrolment. While pre-existing anti-BPSV antibodies did not prevent induction of FMDV-specific neutralizing responses in these animals, the study was not designed to determine how varying levels of baseline vector immunity influence vaccine performance. Future studies in larger cohorts, including both BPSV-seronegative animals (if they can be identified) and animals with differing levels of pre-existing anti-BPSV immunity, will be required to address these questions.

Despite limitations, the present findings provide proof of concept that rBPSV can function as a cattle-adapted vector for *in vivo* production and delivery of FMDV VLPs, inducing neutralizing antibody responses associated with protection despite pre-existing vector immunity. The persistence, reinfection potential and efficient contact transmission of BPSV [10, 15, 25] further raise the possibility that rBPSV vaccines could support repeated antigen exposure through natural re-exposure within cattle populations, thereby reinforcing vaccine-induced immunity.

## Abbreviations

3C^pro^: 3C protease
EPP: expected percentage of protection
FMD: foot-and-mouth disease
FMDV: FMD virus
hpi: h post-infection
hpih post-infection: hours post-infection
LPBE: liquid-phase blocking ELISA
OFTu: ovine foetal turbinate
PPG: Protection against Podal Generalization
rBPSV: recombinant bovine papular stomatitis virus
TCID_50_: 50% tissue culture infectious dose
TEM: transmission electron microscopy
VLPs: virus-like particles
VNT: virus neutralization test
